# Value of endometrial echo pattern transformation after hCG trigger in predicting IVF pregnancy outcome: a prospective cohort study

**DOI:** 10.1186/s12958-019-0516-5

**Published:** 2019-09-05

**Authors:** Zhaojuan Hou, Qiong Zhang, Jing Zhao, Aizhuang Xu, Aihua He, Xi Huang, Shi Xie, Jing Fu, Lan Xiao, Yanping Li

**Affiliations:** 1 Department of Reproductive Medicine, Xiangya Hospital, Central South University, 87 Xiangya Road, Changsha City, Hunan Province 410008 People’s Republic of China; 2 Clinical Research Center For Women’s Reproductive Health In Hunan Province, 87 Xiangya Road, Changsha City, Hunan Province 410008 People’s Republic of China

**Keywords:** Endometrial pattern, Ultrasound, Endometrial secretory transformation, Endometrial receptivity, IVF

## Abstract

**Background:**

There is much value in identifying non-invasive ways of measuring endometrial receptivity, as it has the potential to improve outcomes following in vitro fertilization (IVF). It has been suggested that endometrial echogenicity on the day of hCG administration was a good marker of endometrial receptivity. In the daily practice, we notice that patients with non-homogeneous hyperechoic endometrium on the embryo transfer day usually have lower pregnancy rates. We therefore extended the research onward transformation of echo pattern after hCG trigger to analyze the relationship between endometrial echogenicity transformation and IVF outcomes.

**Methods:**

A total of 146 infertile women undergoing their first IVF cycle were recruited in the prospective cohort study from August 2017 through August 2018. A series of endometrial echo pattern monitoring was carried out in these patients after hCG trigger: hCG day, from 1 through 3 days after ovum pick-up (OPU + 1, OPU + 2, OPU + 3).

**Results:**

The endometrial echogenicity value was calculated as the ratio of the hyperechogenic endometrial area over the whole endometrial area. Clinical pregnancy rate and embryo implantation rate had positive relationship with echogenicity value. The ROC curve analysis of endometrial echogenicity showed the area under curve was greatest on the second day after oocyte retrieval (OPU + 1, 2, 3 were 0.738, 0.765, 0.714 respectively) versus pregnancy. Endometrial echogenicity value on OPU + 2 had a higher predictive efficiency, and the cutoff value was 76.5%. The sensitivity was 61.3% and specificity was 82.0%. When putting the cut-off at <60%, the sensitivity was 93.8% and the specificity was 23.1%.

**Conclusions:**

The endometrial echogenicity value on OPU + 2 was recommended to evaluate endometrial receptivity. It seemed appropriate for clinicians to provide a ‘freeze all’ IVF cycle and transfer in a subsequent frozen-thawed embryos cycle when echogenicity value <60% on OPU + 2.

**Trial registration:**

The registration number was ChiCTR-OOC-17012214 and the registration date was August 1st, 2017.

## Introduction

Endometrium is a highly dynamic tissue undergoing striking physiological changes in response to ovarian steroid hormones. Although ovarian stimulation (OS) can enhance pregnancy rates by increasing the number of oocytes retrieved, the harmful effect of supraphysiologic hormonal levels on endometrial receptivity has been identified [1]. It is commonly accepted that the embryo, the endometrium and the “cross-talk” between these two collaborators account for one-third of implantation failure respectively [2]. The endometrium with normally functioning tissue allows a competent embryo to attach to and invade into it. Therefore, the malfunction of the endometrium is responsible for implantation failure. It is a general opinion that the endometrium is less important than embryos for the final IVF outcome. But the recent data reported by Dr. Brannstrom regarding the IVF clinical pregnancy rate after the embryo transfer in the transplanted uterus were impressive: 100% [3]! All the transplanted uteruses were donated by fertile patients. Thus, the issue may be much more important than believed. There is an urgency to do researches on endometrial receptivity of infertile population.For decades, reproductive scientists have done great jobs to seek for a suitable method to assess endometrial receptivity. Beginning in the 1950s, Noyes proposed the criteria for endometrial dating which was assumed as a possible problem for unsuccessful implantation. ‘Retarded’ endometrium in the luteal phase was reported to have a higher miscarriage rate and lower pregnancy rate than normal development endometrium [4, 5]. Moreover, pinopode scoring may be of value in evaluating endometrial receptivity [6, 7]. In recent years, the use of endometrial receptivity array (ERA) and personalized embryo transfer (pET) in women with recurrent implantation failure (RIF) led to similar ongoing pregnancy rates compared to women with standard embryo transfer [8, 9]. Ultrasound is the widely used non-invasive tool but no consensus on ultrasonic index is reached. Nowadays the commonly used indexes include endometrial pattern [10–12], volume [13, 14], thickness [11, 15, 16], uterine peristalsis [17–19], endometrial and subendometrial blood flows [14, 20, 21].

Endometrial thickness and echo pattern are the most commonly used markers for evaluating endometrial receptivity in clinical practice. However, a meta-analysis showed the predictive ability of endometrial thickness for clinical pregnancy was low as the area under the hierarchical summary receiver operating characteristic (HSROC) curve was only 0.57(95% CI: 0.52–0.61) [22]. Echo pattern is the ultrasonic index reflecting endometrial proliferation and/or stromal decidualization. Previous researches focused on the late proliferative phase in ovarian stimulation cycle [23] and echo pattern was divided into three types: A, B, and C. Pattern A:a triple-line pattern which was consisted of a central hyperechogenic line surrounded by two hypoechoic layers; Pattern B: an isoechogenic pattern in relation to the surrounding myometrium and unclearly central hyperechogenic line; and Pattern C: homogeneous, hyperechogenic endometrium. Endometrium with Pattern A on the day of hCG administration has been extensively reported to have a positive relationship with clinical outcomes [24]. While a Pattern C endometrium seemed to have a detrimental effect on implantation rates [25].

Nevertheless, the effect of echo pattern transformation on embryo implantation outcome was rarely studied. Therefore, we extended the research onward transformation of echo pattern after hCG trigger via monitoring the endometrial echogenicity value on hCG day and the days after ovum pick-up (OPU), and to analyze the relationship between echo pattern transformation and IVF pregnancy outcomes. According to our research, endometrial echogenicity value on OPU + 2 was found to have a higher predictive efficiency, and the cutoff value was 76.5% with the sensitivity was 61.3% and specificity was 82.0%. It seemed judicious for physicians to freeze the embryos and transfer them in a subsequent thaw cycle when echogenicity value<60% on OPU + 2.

## Materials and methods

### Study design

Prospective cohort study involving ultrasound examination on hCG day and the days after ovum pick-up of women undergoing their first in vitro fertilization and fresh embryo transfer.

### Study population

Patients were recruited from the Reproductive Medicine Center of Xiangya Hospital of Central South University (Changsha, China) from August 2017 through August 2018. The baseline data, such as age, body mass index (BMI) and duration of infertility, were collected by our clinic staff.

Inclusion criteria: (1) Age<42 years; (2) Morphologically normal uterine (no intrauterine adhesions, submucosal fibroids distorting the uterine cavity, adenomyosis and polyps) which was confirmed by hysteroscopy or ultrasonography; (3) At least one top-quality embryo [26] transferred.

Exclusion criteria: elevated plasma progesterone level>1.5 ng/ml or plasma E2 level > 5000 pg/ml on the day of hCG administration, and the number of oocytes retrieved≥20. These patients were excluded because of the cancellation of their fresh ET cycle. Subjects with a medioverted uterus that does not adequately display the ultrasonic texture of endometrium were also excluded.

### Ovarian stimulation and IVF-ET procedures

The stimulation regimen was personalized and mainly based on maternal age and ovarian reserve function. Controlled ovarian hyperstimulation was performed when serum estradiol concentration (E2) level ≤ 50 pg / ml, maximum follicle diameter < 10 mm with no ovarian cysts. Exogenous high purified (HP) FSH (Lishenbao; LiZhu, Zhuhai, China) and/or hMG (Lebaode; LiZhu, Zhuhai, China) was injected to induce follicular development. The initial dosage of gonadotrophin ranged from 112.5 to 300 IU/day and was adjusted later according to the speed of follicular growth during treatment. Final maturation of oocytes was induced with 6000 IU − 10,000 IU of hCG (Rongcuxingsu; LiZhu, Zhuhai, China), when 2–3 leading follicles ≥18 mm in diameter and serum E2 level was in accordance with the number of dominant follicles, oocytes were then retrieved 36 h later under the guidance of transvaginal ultrasonography (TVS). One or two embryos were transferred 48 or 72 h after follicle aspiration according to the condition of embryos. All the embryo transfers were done by a single professor. The luteal phase was supported by oral progesterone capsules (Qining; Aisheng, Hangzhou, China) at a dose of 200 mg per day and vaginal micronised progesterone (Utrogestan; Besins-Iscovesco Pharmaceuticals, Paris, France) at a dose of 400 mg per day starting on the day of egg retrieval. Clinical pregnancy was confirmed by a gestational sac with or without cardiac activity at approximately 6 weeks gestation visualized on transvaginal ultrasound. Implantation rate (IR) was defined as the number of gestational sacs divided by the number of transferred embryos.

### Ultrasound measurement and image analysis

We performed transvaginal ultrasound on the day of hCG administration and 1 through 3 days after ovum pick-up (OPU + 1, OPU + 2, OPU + 3). The uterus was displayed on a sagittal plane using a 8-mHZ transduser (GE Voluson E10, the United States) by clinicians. Measurements were avoided during the myometrium contractions. A total of 566 sonograms from 146 patients were stored and analyzed with ImageJ software (version1.8.0) using its measure and threshold function.

The endometrial echogenicity value was calculated as the ratio of the hyperechogenic endometrial area over the whole endometrial area. On the basis of previously published research, the minimal gray value of hyperechogenic endometrium was defined as the gray value within the endometrial border which exceeded 10% of the mean gray value of the whole surrounding myometrium, that is, 1.1 times of the mean gray value of the myometrium [27, 28]. The mean gray value of the myometrium was obtained via the measure function of ImageJ software. The endometrial echogenicity value was carried out by the threshold function of setting the minimal gray value of hyperechoic endometrium. The endometrial borders and myometrial borders were manually portrayed using the freehand selections function. The endometrial thickness was recognized as its maximum distance between the anterior and posterior wall of the endometrium-myometrium interfaces. This study had good inter-rater reliability with intraclass correlation coefficient greater than 0.9.

The endometrial echogenicity transformation was compared according to pregnant status: pregnant group and non-pregnant group. And for the purpose of simplifying the association between endometrial echogenicity transformation and IVF outcome, the cycles were sorted into five echogenicity groups: ≤60%, 61–70%, 71–80%, 81–90%, and>90%.

### Statistical analysis

Quantitative variables were expressed as mean ± SD or as median (range) according to distribution. Categorical data was presented as counts and percentages. Intraclass correlation coefficient (ICC) was used to measure the inter-rater reliability. Statistical inference of data on demographic, controlled ovarian hyperstimulation (COH), and embryology between pregnant and non-pregnant group were compared with Student’s t-test or Fisher’s exact test. Endometrial thickness and echogenicity value on HCG day and OPU + 1, 2, 3 in a longitudinal timeline were analyzed using analysis of variance for repeated measurement data. Endometrial echogenicity value between pregnant and non-pregnant women were compared using paired Student’s t-test according to their age, stimulation protocol, the number of embryos transferred and day of embryo transfer. The clinical pregnancy rate and implantation rate on OPU + 1, 2, 3 between five different echogenicity groups were compared by trend chi-square test. The values of endometrial echogenicity on the days of OPU + 1, 2, 3 were analyzed using receiver operating characteristic (ROC) curve analysis to quantify the optimal cut-off point to determine if this parameter predicted reproductive outcome. Further, a ROC curve analysis was used to assess the predictive capacity of the combination of echogenicity value and thickness. P<0.05 was considered statistically significant. All statistical tests were performed using SAS (version 9.4), SPSS version 25.0 (SPSS, Chicago, IL).

### Ethical approval

This study was conducted according to the Declaration of Helsinki for Medical Research and was approved by the Ethics Committee of the Reproductive Medicine Center of Xiangya Hospital and registered with Chinese Clinical Trial Registry (http://www.chictr.org.cn; registration number: ChiCTR-OOC-17012214). Written informed consent was obtained from all patients prior to study participation.

## Results

### Overall data

The clinical indications for IVF-ET were tubal factors (86.3%), male factors (6.2%), multiple factors (6.2%), ovulation disorder (0.7%) and idiopathic infertility (0.7%). The data of 17 cases were missing on OPU + 3 day. 11 of them were due to Day-2 embryo transfer, and the remaining 6 of them were not collected. Besides, one data was not collected on HCG day. Of the 5 women with Pattern C endometrium on HCG day, one achieved clinical pregnancy with morula.

A total of 271 embryos were transferred into 146 subfertile patients in 146 fresh IVF-ET cycles. The maternal age ranged from 20 to 41 years (mean ± SD:30.12 ± 4.31 years), and mean(±SD) BMI was 21.24 ± 2.64 Kg*m^− 2^. Baseline plasma FSH, LH and E2 were within the normal range, at 6.97 ± 1.95 mIU/mL, 6.09 ± 3.48 mIU/mL and 39.21 ± 22.81 pg/mL, respectively. Ovarian reserve testing showed patients with good ovarian reserve function, with AMH was 3.97 ± 2.75 ng/mL and antral follicle count was 14.38 ± 5.94. The ovarian stimulation lasted 11.38 ± 2.02 days and 2082.72 ± 776.52 IU gonadotrophin were administered to patients. On the day of hCG administration, serum E2 levels reached 2548.80 ± 1169.68 pg/mL, serum LH and progesterone profiles were 1.66 ± 1.01 mIU/mL and 0.64 ± 0.32 ng/mL, respectively. 10.99 ± 4.08 oocytes were retrieved and top-quality embryo rate was 0.51 ± 0.24. 1.86 ± 0.35 embryos were transferred to the subjects. The mean(±SD) endometrial echogenicity values were 0.43 ± 0.14(43% of the whole endometrial surface), 0.71 ± 0.14, 0.74 ± 0.13 and 0.78 ± 0.13 respectively on hCG day, OPU + 1, OPU + 2 and OPU + 3, which increased significantly in a longitudinal timeline(*P* < 0.001). A stepwise increase of endometrial thickness was observed on hCG day, OPU + 1, OPU + 2 and OPU + 3(11.24 ± 2.73 mm,11.68 ± 3.06 mm,12.35 ± 3.46 mm and 12.48 ± 3.40 mm, respectively. P < 0.001). Clinical pregnancy rate (CPR) and the implantation rate were 55.5% (81 out of 146) and 39.5% (107 out of 271), respectively.

### Data on demographic, COH and embryology

The demographic, COH and embryology data are given in Table 1. There was no significant difference in the age of women between pregnant and non-pregnant group (29.70 ± 4.19 years and 30.63 ± 4.44 years, respectively). The duration of infertility (3.84 ± 2.70 years and 3.81 ± 3.11 years, respectively) and body mass index (BMI, 21.14 ± 2.49 Kg*m^− 2^ and 21.37 ± 2.84 Kg*m^− 2^, respectively) were similar between the two groups. With regard to ovarian reserve function, including basal FSH profile (6.76 ± 1.91mIU/mL vs 7.22 ± 1.97 mIU/mL), Anti-Mullerian Hormone (4.10 ± 2.73 ng/mL vs 3.82 ± 2.78 ng/mL), number of antral follicles (14.46 ± 6.34 vs 14.29 ± 5.45), no statistically significant differences were found in the two groups. When it comes to controlled ovarian stimulation (COS), the length of stimulation (11.35 ± 1.94 days vs 11.43 ± 2.12 days), total dose of gonadotrophin administered (2020.70 ± 789.82 IU vs 2160.00 ± 758.56 IU), serum LH levels (1.75 ± 1.03mIU/mL vs 1.53 ± 0.98mIU/mL), E2 levels (2585.65 ± 1177.69mIU/mL vs 2503.44 ± 1167.27 mIU/mL) and progesterone levels (0.67 ± 0.33 ng/ml vs 0.59 ± 0.30 ng/ml) at the trigger day, number of oocytes retrieved (10.83 ± 3.97 vs 11.20 ± 4.24), top-quality embryo rate (0.53 ± 0.25 vs 0.50 ± 0.24) and number of embryos transferred (1.90 ± 0.30 vs 1.80 ± 0.40) were comparable between pregnant and not pregnant women.

**Table 1:** Demographic, COH and embryology data in the two groups

Characteristics	Pregnant^a^ *n* = 81	Non-pregnant *n* = 65	*P*
Age(y)	29.70 ± 4.19	30.63 ± 4.44	0.197
Etiology of infertility			0.292
Tubal *n*(%)	67 (82.7%)	59 (90.8)	
Male *n*(%)	6 (7.4%)	3 (4.6)	
Both *n*(%)	7 (8.6%)	2 (3.1)	
Idiopathic *n*(%)	1 (1.2%)	0 (0%)	
Ovulation disorder *n*(%)	0 (0%)	1 (1.5)	
BMI (Kg*m^−2^)	21.14 ± 2.49	21.37 ± 2.84	0.598
Duration of infertility(y)	3.84 ± 2.70	3.81 ± 3.11	0.947
Basal FSH (mIU/mL)	6.76 ± 1.91	7.22 ± 1.97	0.161
Basal LH (mIU/mL)	6.11 ± 3.24	6.06 ± 3.77	0.945
Basal E2 (pg/mL)	40.78 ± 23.60	37.30 ± 21.83	0.368
AMH (ng/mL)	4.10 ± 2.73	3.82 ± 2.78	0.557
AFC, *n*	14.46 ± 6.34	14.29 ± 5.45	0.866
Length of stimulation (days)	11.35 ± 1.94	11.43 ± 2.12	0.801
Total dose of Gn administered (IU)	2020.70 ± 789.82	2160.00 ± 758.56	0.283
^b^LH levels (mIU/mL)	1.75 ± 1.03	1.53 ± 0.98	0.191
^b^E2 levels (pg/mL)	2585.65 ± 1177.69	2503.44 ± 1167.27	0.675
^b^P levels (ng/mL)	0.67 ± 0.33	0.59 ± 0.30	0.116
No. of oocytes retrieved	10.83 ± 3.97	11.20 ± 4.24	0.584
Top-quality embryo rate	0.53 ± 0.25	0.50 ± 0.24	0.406
No. of embryos transferred	1.90 ± 0.30	1.80 ± 0.40	0.095

*BMI* Body mass index, *E2* Oestradiol, *AMH* Anti-Mullerian hormone, *AFC* Antral follicle count, *Gn* Gonadotrophin

^a^Patients who achieved a clinical pregnancy

^b^ on the day of hCG administration

### Data on endometrial echogenicity values

**Fig. 1:**
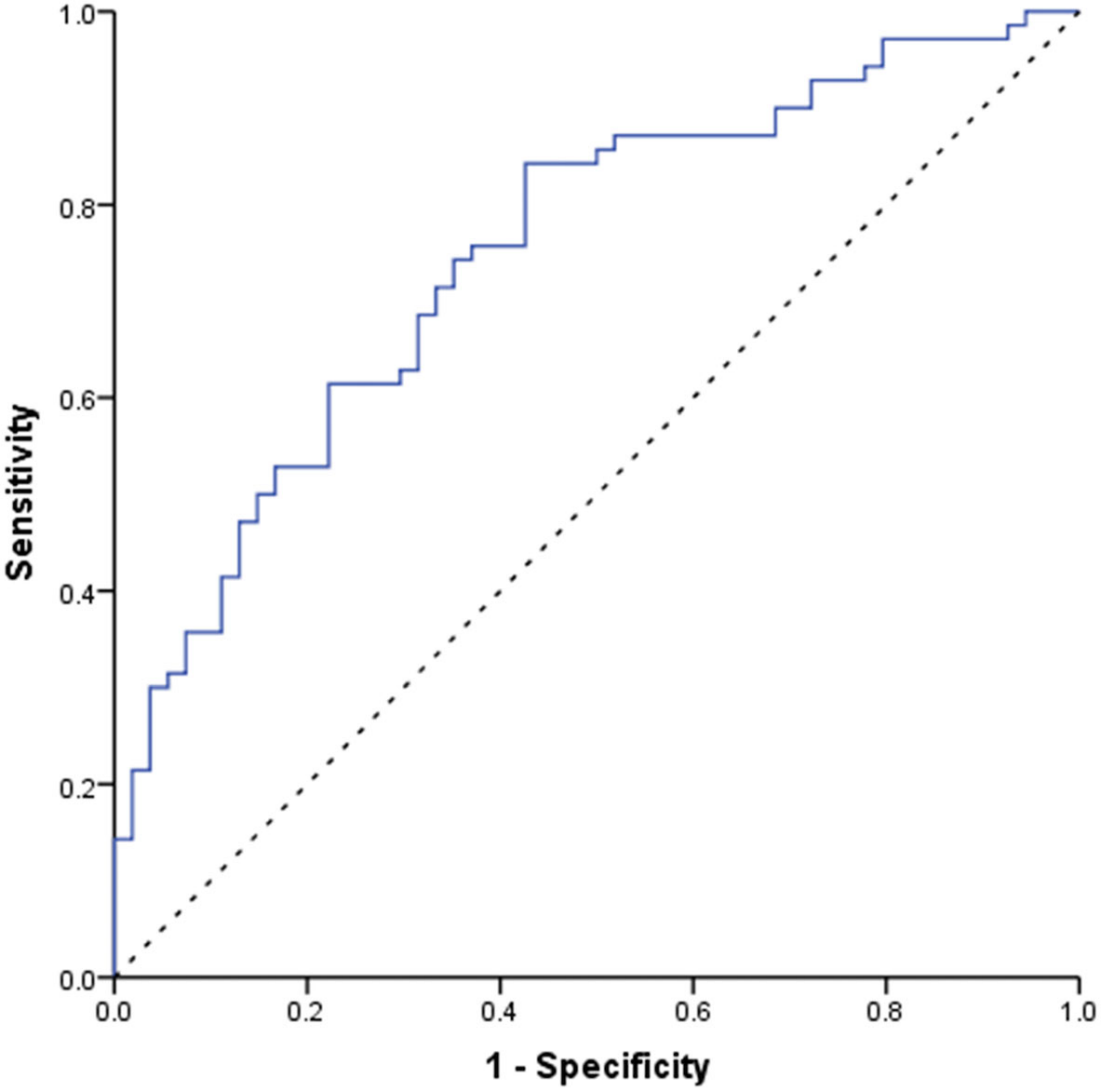
Endometrial echogenicity in infertile women after hCG trigger during COH cycles. (A) Endometrial echogenicity with non-pregnant women. (B) Endometrial echogenicity with pregnant women. The black arrow points to the endometrium-myometrium interfaces. a, HCG day; b, OPU + 1; c, OPU + 2; d, OPU + 3

The endometrial echogenicity values differed markedly between pregnant and non-pregnant group on OPU + 1 (0.75 ± 0.12 vs 0.67 ± 0.14, *P* < 0.001), OPU + 2 (0.78 ± 0.12 vs 0.68 ± 0.14, P < 0.001) and OPU + 3 (0.81 ± 0.11 vs 0.72 ± 0.14, P < 0.001), which were demonstrated in Table 2 and Fig. 1. In addition, there was no significant difference concerning the endometrial echogenicity values on hCG day (0.45 ± 0.14 vs 0.45 ± 0.17, *P* = 0.888) between the two groups.

**Table 2:** Endometrial echogenicity values after hCG trigger in the two groups

Group	N	HCG	OPU + 1	OPU + 2	OPU + 3
Pregnant^a^	65	0.45 ± 0.14	0.75 ± 0.12	0.78 ± 0.12	0.81 ± 0.11
Non-pregnant	65	0.45 ± 0.17	0.67 ± 0.14	0.68 ± 0.14	0.72 ± 0.14
P		0.888	< 0.001	< 0.001	< 0.001

^a^Patients who achieved a clinical pregnancy

**Fig. 2:**
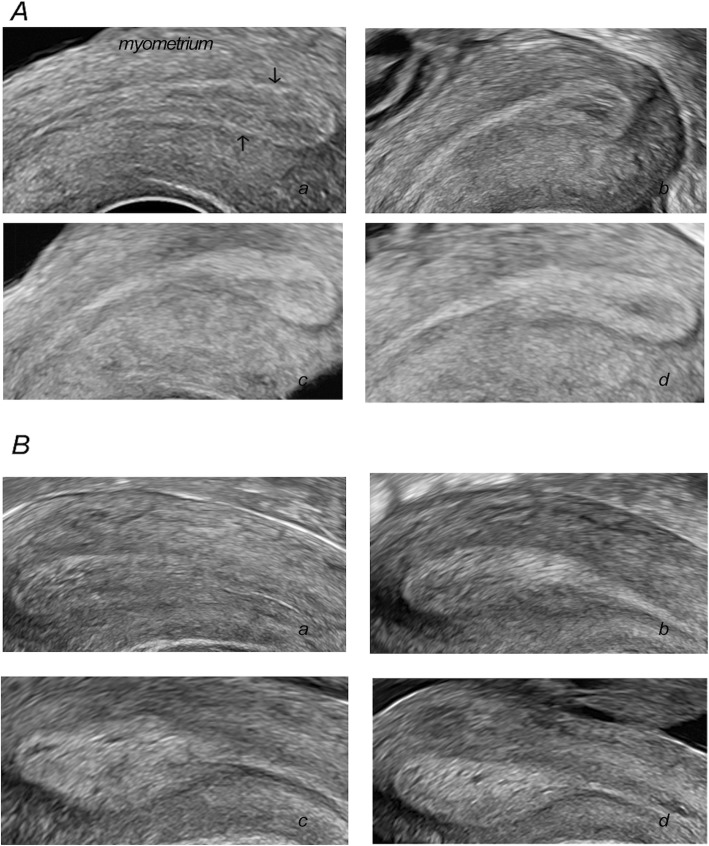
Clinical pregnancy rates in different endometrial echogenicity groups assessed on OPU + 1, OPU + 2 and OPU + 3

**Fig. 3:**
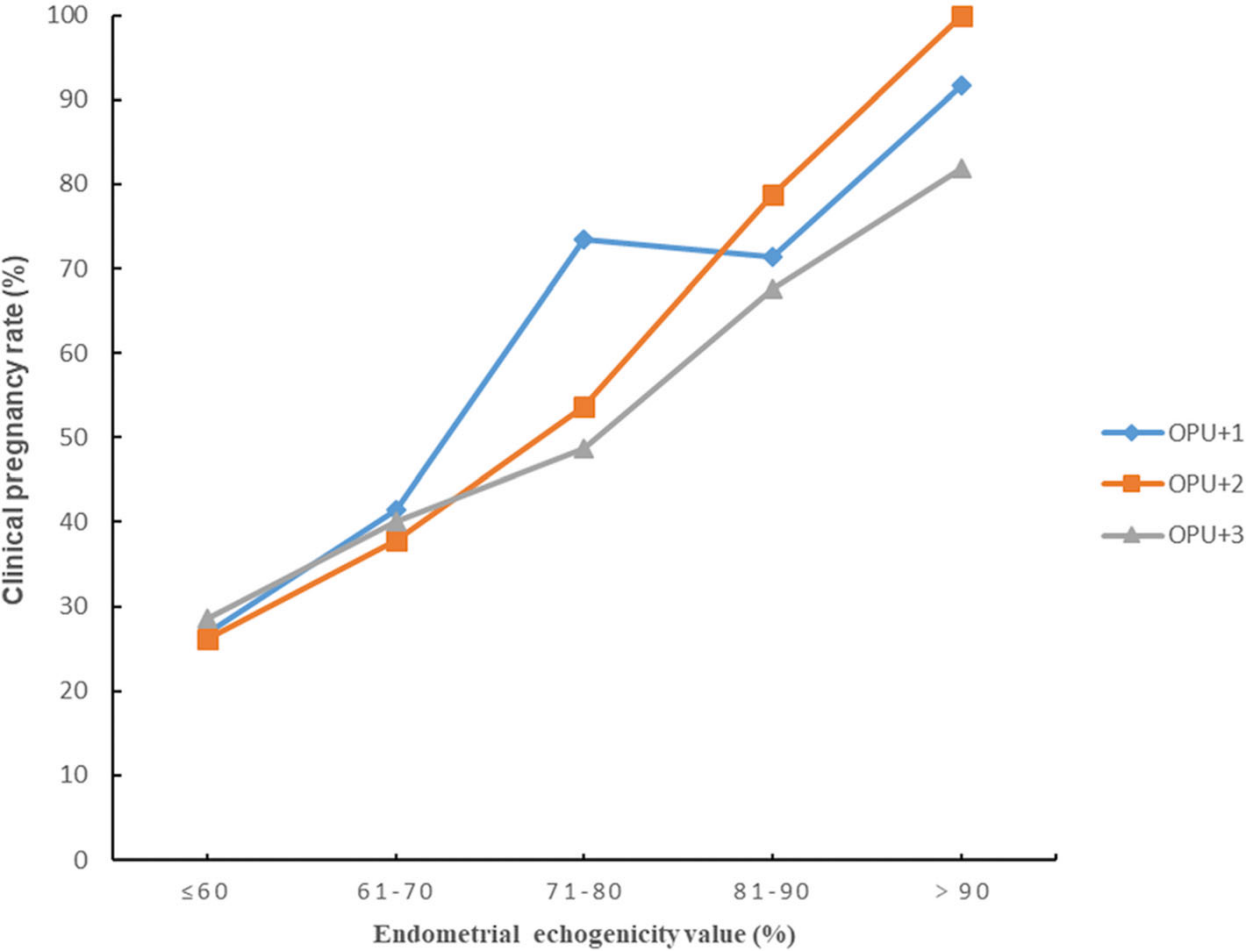
Implantation rates in different endometrial echogenicity groups assessed on OPU + 1, OPU + 2 and OPU + 3

For further analysis, we divided the endometrial echogenicity values into five groups: ≤60%, 61–70%, 71–80%, 81–90%, and>90%. There were 27, 42, 34, 29 and 14 cycles in the five echogenicity groups on OPU + 1 respectively. There were 24, 29, 42, 34 and 17 cycles in the five echogenicity groups on OPU + 2 respectively. There were 15, 15, 41, 36 and 22 cycles in the five echogenicity groups on OPU + 3 respectively. Figure 2 displayed the clinical pregnancy rate increased significantly from the lowest to the highest endometrial echogenicity groups on OPU + 1, OPU + 2 and OPU + 3. There was an identical trend for embryo implantation rate as revealed in Fig. 3.

**Fig. 4:**
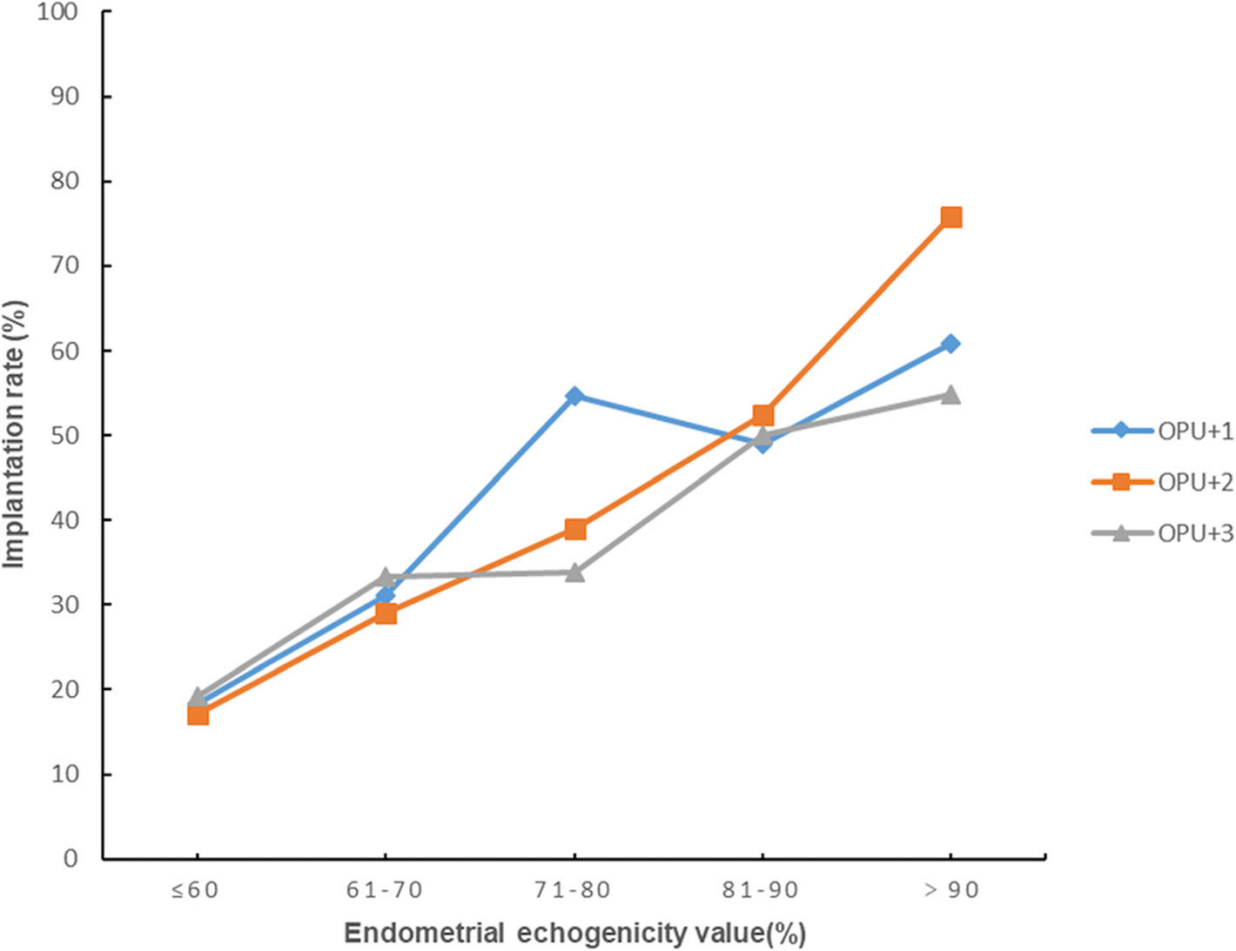
ROC curve of endometrial echogenicity value on OPU + 1,2,3 for successful clinical pregnancy. The areas under the ROC curve were 0.738(95%CI:0.656–0.819), 0.765(95%CI: 0.688–0.842), 0.714(95%CI:0.624–0.804) respectively on OPU + 1, OPU + 2 and OPU + 3. Endometrial echogenicity value on OPU + 2 had the most predictive value, and the cutoff value was 76.5%. The sensitivity was 61.3% and the specificity was 82.0%

As illustrated in Fig. 4, the ROC curve analysis of endometrial echogenicity on OPU + 1, OPU + 2 and OPU + 3 showed areas under the curve (AUC) were 0.738(95%CI:0.656–0.819), 0.765(95%CI: 0.688–0.842), 0.714(95%CI:0.624–0.804), respectively. The potential cut-off points at 0.705, 0.765, 0.785, respectively on OPU + 1, OPU + 2 and OPU + 3 had a sensitivity of 70.0, 61.3, 67.1%, respectively and a specificity of 70.5, 82.0, 68.5%, respectively. The sensitivity was 91.4, 93.8 and 94.4% respectively and the specificity was 27.7, 23.1 and 13.8% respectively on OPU + 1,2,3 when putting the cut-off at < 60%.

**Fig. 5:**
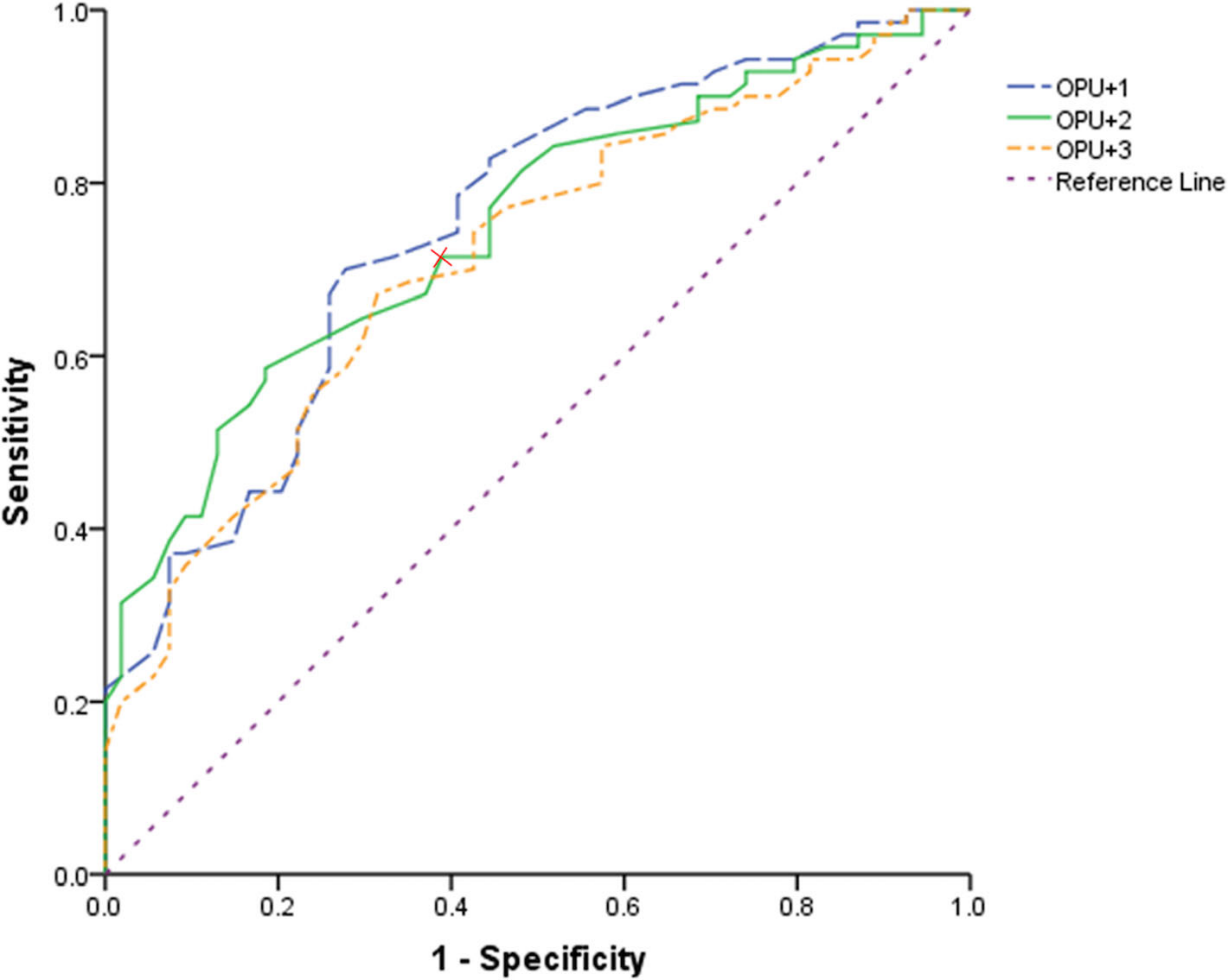
ROC curve of the combination of endometrial echogenicity value on OPU + 2 and thickness on OPU + 3. The areas under the ROC curve was 0.751 (95%CI:0.665–0.836)

Moreover, the ROC curve analysis of endometrial thickness on OPU + 1, OPU + 2 and OPU + 3 showed areas under the curve were 0.523(95%CI:0.428–0.618), 0.536(95%CI: 0.441–0.631), 0.580(95%CI:0.481–0.680), respectively. Therefore, endometrial echogenicity value on OPU + 2 and thickness on OPU + 3 were combined to assess the predictive capacity of pregnancy and this information was shown on Fig. 5. The AUC was 0.751 (95%CI: 0.665–0.836) which was lower than AUC solely determined by echogenicity value on OPU + 2.

## Discussion

Our results indicate that OPU + 2 had a higher predictive efficiency and the more hyperechoic the endometrium was after hCG trigger, the higher the clinical pregnancy rate and implantation rate were. The high proportion of tubal infertility in our study may attribute to the increasing amount of artificial abortion in China nowadays. The one who obtained successful pregnancy with Pattern C endometrium on the day of hCG administration denoted that other factors, possibly linked to embryo quality, may mitigate the detrimental impact of reduced endometrial receptivity to some extent. The endometrial echo values on OPU + 1, OPU + 2 and OPU + 3 were approximately 10% higher in the clinical pregnant group than in the non-pregnant group, this may because a fully developed luteal phase endometrium is more receptive for implanting blastocyst to adhere. There appeared to be a steep increase of CPR and IR in the ≥71% endometrial echogenicity group on OPU + 1 day and in the ≥81% endometrial echogenicity group on days of OPU + 2 and OPU + 3. Accordingly, if the endometrial secretory transformation rate could be hastened to more than 71% on OPU + 1 day and more than 81% on OPU + 2 and OPU + 3 day, the reproductive outcomes may get huge improvements. Clinically, endometrial pattern information and endometrial thickness information are often considered together. However, in the present study, a suggestion of combining endometrial echogenicity value and thickness to predict pregnancy could not be made since the AUC of combining the two factors was lower than that of echogenicity value.

Several characteristics differentiate our study from other investigations. First, we quantitatively explored the average value of endometrial echo pattern transformation after HCG trigger in the COH cycle, and detected the optimal threshold of endometrial echo values that could maximize the chance of CPR and IR, while former studies used ambiguous evaluation method of three classified types of endometrial pattern to correlate endometrial ultrasonic texture with clinical outcomes. The method of assessing sonographic endometrial texture in our study finely avoids the disparity of every sonographer’s interpretation. Furthermore, what we highlighted was the serially process of luteal endometrial development in the COH cycle as opposed to HCG day or only the embryo transfer day assessed in the luteal phase that have been extensively reported. And from the serial measurements, we picked up OPU + 2 as an ideal point to assess endometrial receptivity.

One limitation of our investigation was the lack of blood sampling on circulating levels of steroid hormones. Thomsen et al. reported that the optimal pregnancy chance was achieved when the serum progesterone level was 60–100 nmol / L in the early luteal phase, and the optimal progesterone level in the mid-luteal phase was 150–250 nmol / L, although the progesterone threshold in this study was only applicable to the IVF and fresh embryo transfer cycle supported by vaginal route [29]. Comprehensive evaluation of luteal endometrial echogenicity values and plasma progesterone levels should be combined to make better clinical decisions in the future study. And although the calculation made by ImageJ may be objective, manual border selection was subjective, thus personal bias was inevitable. However, intraclass correlation coefficient showed that there was good inter-rater reliability towards our measurements.

In the early history of assisted reproductive technology (ART), a number of researchers have made effort to depict the cyclic changes of endometrium on ultrasonography by correlating with its histomorphologic findings: a thin echogenic interface is observed during the menstrual phase, the endometrium thickens gradually and the functionalis becomes hypoechoic or isoechogenic throughout the early-mid proliferative phase, a “triple-line” sign in the late proliferative phase, and then the echogenicity increases from the basal layer to the functional layer and finally encompassing the entire endometrial lumen [30, 31]. It is commonly accepted that the hyperechogenic middle line represents the uterine cavity, and the remaining two hyperechogenic line are associated with the endometrium-myometrium interfaces, while there is still controversy towards the leading cause of hyperechogenic texture of secretory endometrium. Fleischer et al. speculated that the hyperechoic texture of secretory endometrium was related to the increased storage of echogenic mucin and glycogen in the distended and tortuous endometrial glands [30], whereas Grunfeld et al. hold the view that the homogeneous hyperechoic endometrium in the late secretory phase might indicate the stromal edema after comparing the endometrial chronological date with glandular histology and stromal histology respectively [32].

The process of endometrial transformation from proliferative phase to secretory phase under the steroids hormonal milieu is called endometrial decidualization. The impairment of decidualization is associated with infertility, recurrent spontaneous abortion (RSA) and intrauterine fetal growth restriction. A variety of biological substances, including hormones, cytokines, immune cells and signaling pathways, are involved in the network regulation mode of decidualization.

The cyclic changes of endometrium is regulated by ovarian hormones and its receptors, and the endometrial luteal phase development may alter in IVF cycle due to the supraphysiological hormone levels. In COH cycle, the degree of endometrial echogenicity grew faster on the days of oocyte retrieval and ET in the high progesterone group(P>0.9 ng/ml at trigger day) than in the low progesterone group while it appeared similar on the day of hCG administration [27]. Supraphysiological progesterone levels could increase blood progesterone concentration and enhance endometrial secretory transformation in artificial hormonal replacement cycle. Conducting biopsy on Day 24 dated morphologically to Day 25.3 ± 0.4 denoted an advanced maturation of stroma [33]. Similarly, high progesterone receptors in the pre-ovulatory phase promoted endometrial responsiveness to progesterone stimulation after ovulation [34]. Human uterine natural killer (uNK) cells were abundant in the late secretory phase and sustained till first trimester pregnancy suggesting that they had an essential role in the progesterone-dominant phase. Studies of endometrial sampling indicated that uNK cells contributed to angiogenic process and circulatory regulation [35]. A recent study reported that the integrin-linked kinase played a pivotal part in morphologic transformation of endometrial stromal cells. It may function through organization of actin cytoskeleton or GSK3β signaling pathway [36]. There is no convincing evidence for clinical application emerged from these studies. Apart from the mechanisms outlined above, it is important to know that based upon the full development of proliferative phase, a satisfying secretory phase of endometrial transformation can occur. Moreover, it should be noticed that the cause for defective endometrial secretory transformation is heterogeneous and any impaired point in the regulation process can lead to female infertility and failure of blastocyst implantation. However, the methodology used in our study is a non-invasive and simple way to recognize defective endometrial secretory transformation in daily practice. Thereby, clinicians can give patients more appropriate embryo transfer recommendations and reduce the economic loss and depression resulting from implantation failure. Based on our research, we recommend the second day after oocyte retrieval as a time-point for patient ideally undergo ultrasound to assess receptivity instead of serial measurements. When echogenicity value<60% on OPU + 2, it seemed advisable for clinicians to freeze the embryos from the present cycle and transfer them in a subsequent thaw cycle.

## In conclusion

The endometrial echo pattern transformation after hCG trigger in COH cycle significantly related to clinical outcomes. Endometrial echogenicity value <60% on OPU + 2 was associated with an extremely low CPR and IR, so cancellation of fresh ET cycle might be a wise alternative for both physicians and patients. Further investigation is required to elaborate the key mechanism behind the regulation of endometrial secretory transformation and to adopt treatment to accelerate the appearance of ultrasonic hyperechoic endometrium after oocyte retrieval.

## Data Availability

The datasets used and/or analysed during the current study are available from the corresponding author on reasonable request.

## Abbreviations

ART: Assisted reproductive technology
AUC: Areas under the curve
BMI: Body mass index
COH: Controlled ovarian hyperstimulation
COS: Controlled ovarian stimulation
CPR: Clinical pregnancy rate
ERA: Endometrial receptivity array
HP: High purified
HSROC curve: Hierarchical summary receiver operating characteristic curve
ICC: Intraclass correlation coefficient
IR: Implantation rate
IVF: In vitro fertilization
OPU + 1: One day after ovum pick-up
OPU + 2: Two days after ovum pick-up
OPU + 3: Three days after ovum pick-up
OPU: Ovum pick-up
OS: Ovarian stimulation
pET: Personalized embryo transfer
RIF: Recurrent implantation failure
ROC: Receiver operating characteristic
RSA: Recurrent spontaneous abortion
TVS: Transvaginal ultrasonography
uNK cells: Uterine natural killer cells
