# Correction to: Value of endometrial echo pattern transformation after hCG trigger in predicting IVF pregnancy outcome: a prospective cohort study

**DOI:** 10.1186/s12958-020-00647-3

**Published:** 2020-09-08

**Authors:** Zhaojuan Hou, Qiong Zhang, Jing Zhao, Aizhuang Xu, Aihua He, Xi Huang, Shi Xie, Jing Fu, Lan Xiao, Yanping Li

**Affiliations:** 1 Department of Reproductive Medicine, Xiangya Hospital, Central South University, 87 Xiangya Road, Changsha City, Hunan Province 410008 People’s Republic of China; 2 Clinical Research Center For Women’s Reproductive Health In Hunan Province, 87 Xiangya Road, Changsha City, Hunan Province 410008 People’s Republic of China

**Correction to: Reprod Biol Endocrinol 17, 74 (2019)**

**https://doi.org/10.1186/s12958-019-0516-5**

Following publication of the original article [1], the authors reported an error in the order of the figures.

The Fig. 1 published is supposed to be Fig. 5. The Fig. 2 published is supposed to be Fig. 1. The Fig. 3 published is supposed to be Fig. 2. The Fig. 4 published is supposed to be Fig. 3. The Fig. 5 published is supposed to be Fig. 4. The correct order of the figures are as follows:

**Fig. 1:**
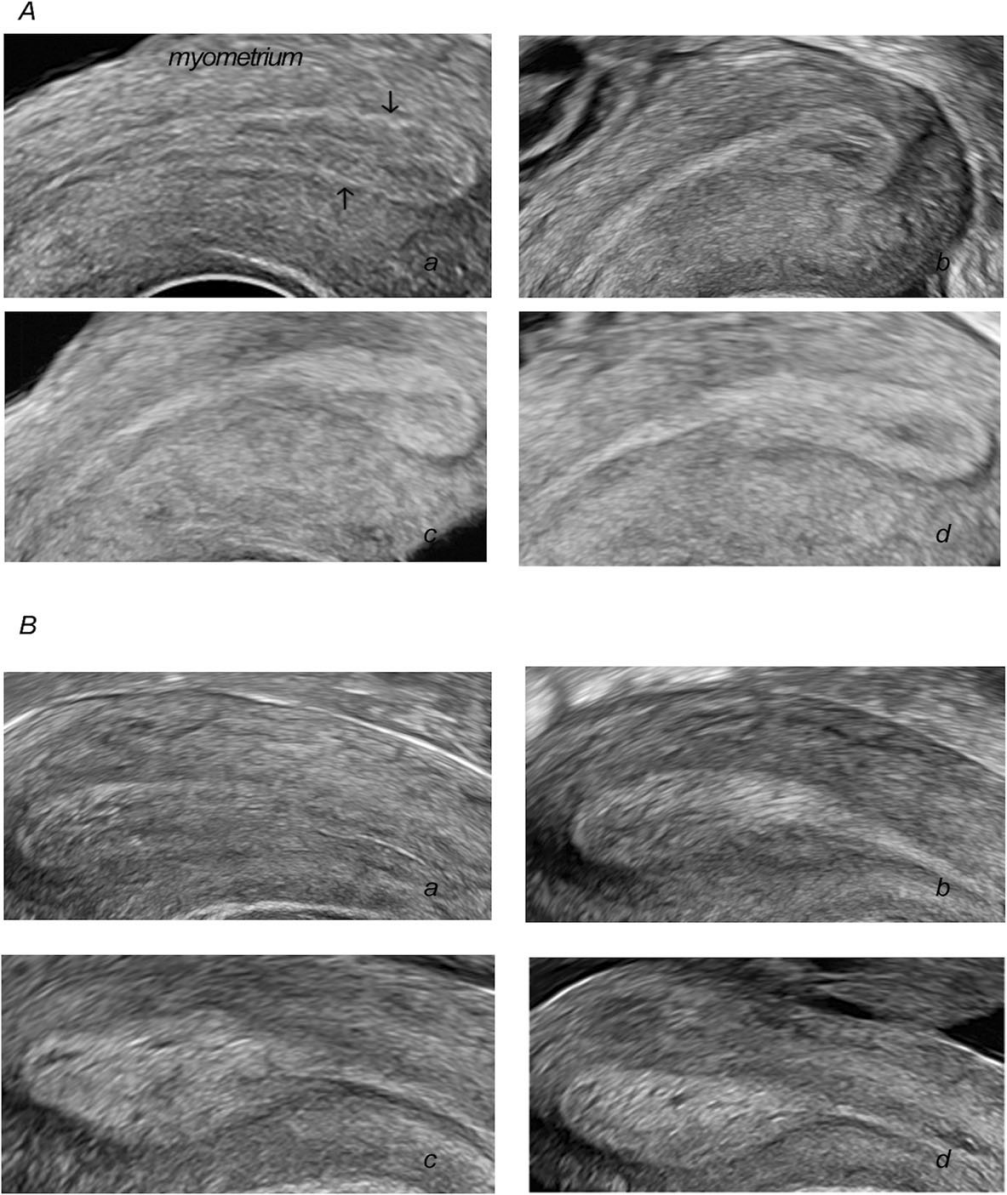
Endometrial echogenicity in infertile women after hCG trigger during COH cycles. (A) Endometrial echogenicity with non-pregnant women. (B) Endometrial echogenicity with pregnant women. The black arrow points to the endometrium-myometrium interfaces. a, HCG day; b, OPU + 1; c, OPU + 2; d, OPU + 3

**Fig. 2:**
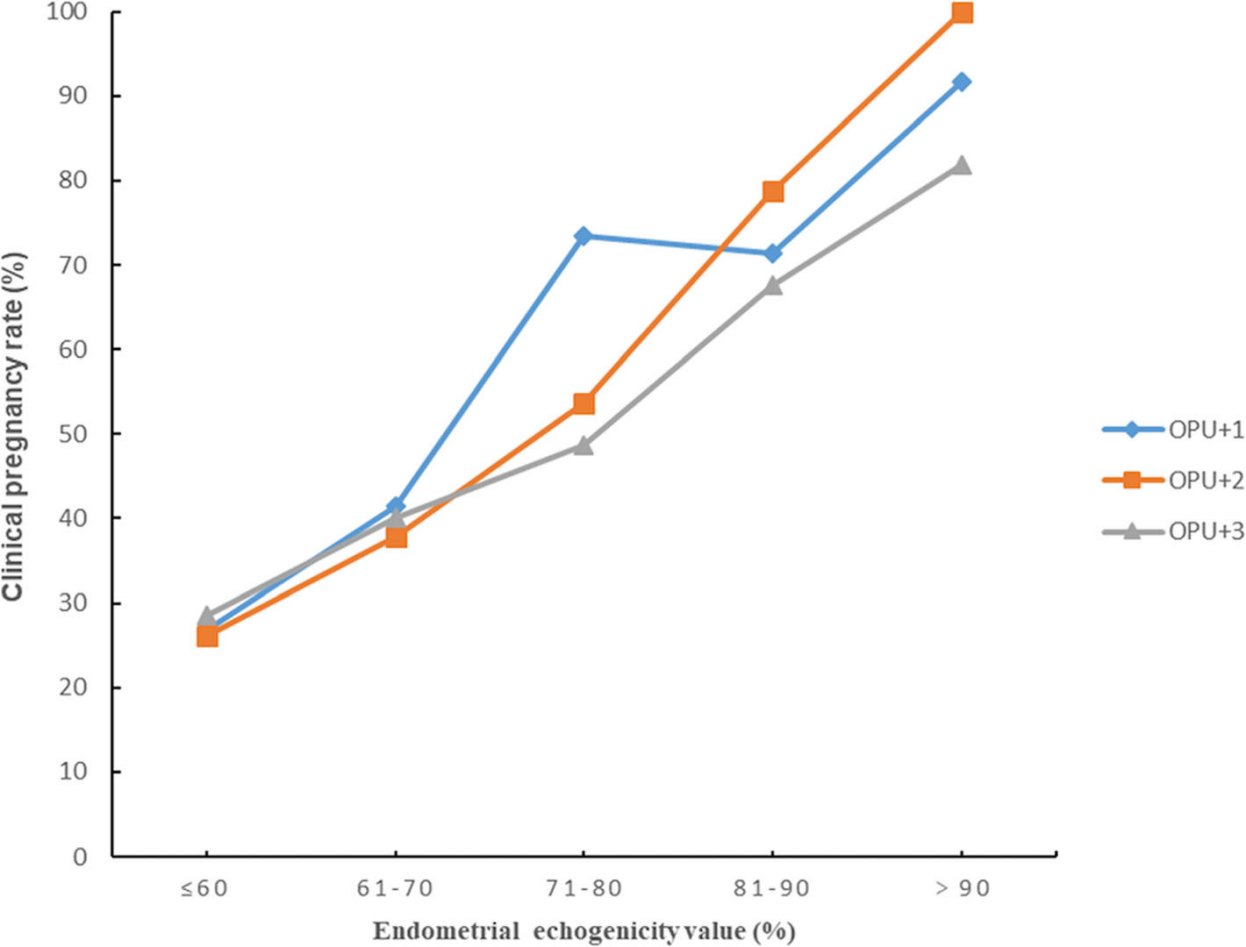
Clinical pregnancy rates in different endometrial echogenicity groups assessed on OPU + 1, OPU + 2 and OPU + 3

**Fig. 3:**
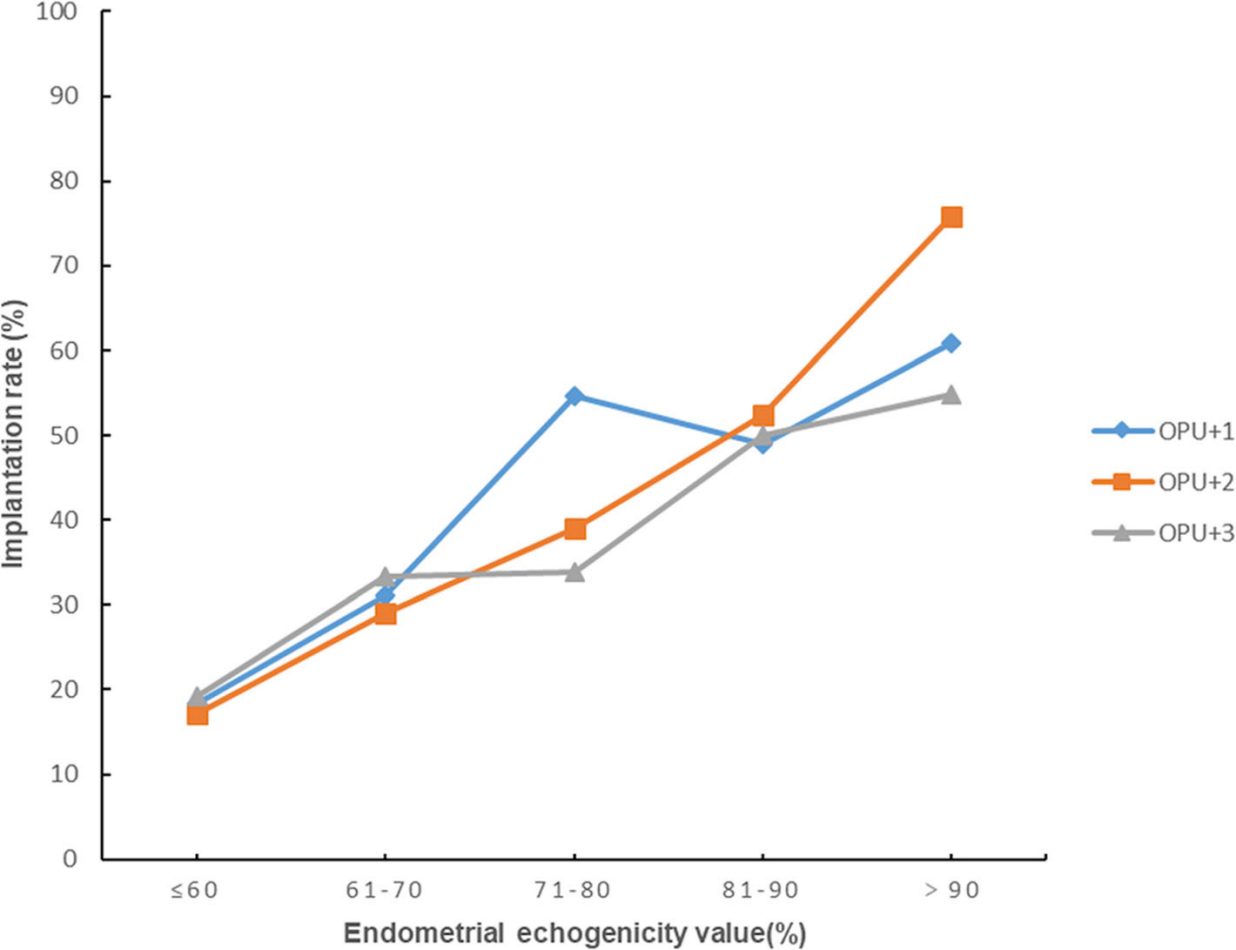
Implantation rates in different endometrial echogenicity groups assessed on OPU + 1, OPU + 2 and OPU + 3

**Fig. 4:**
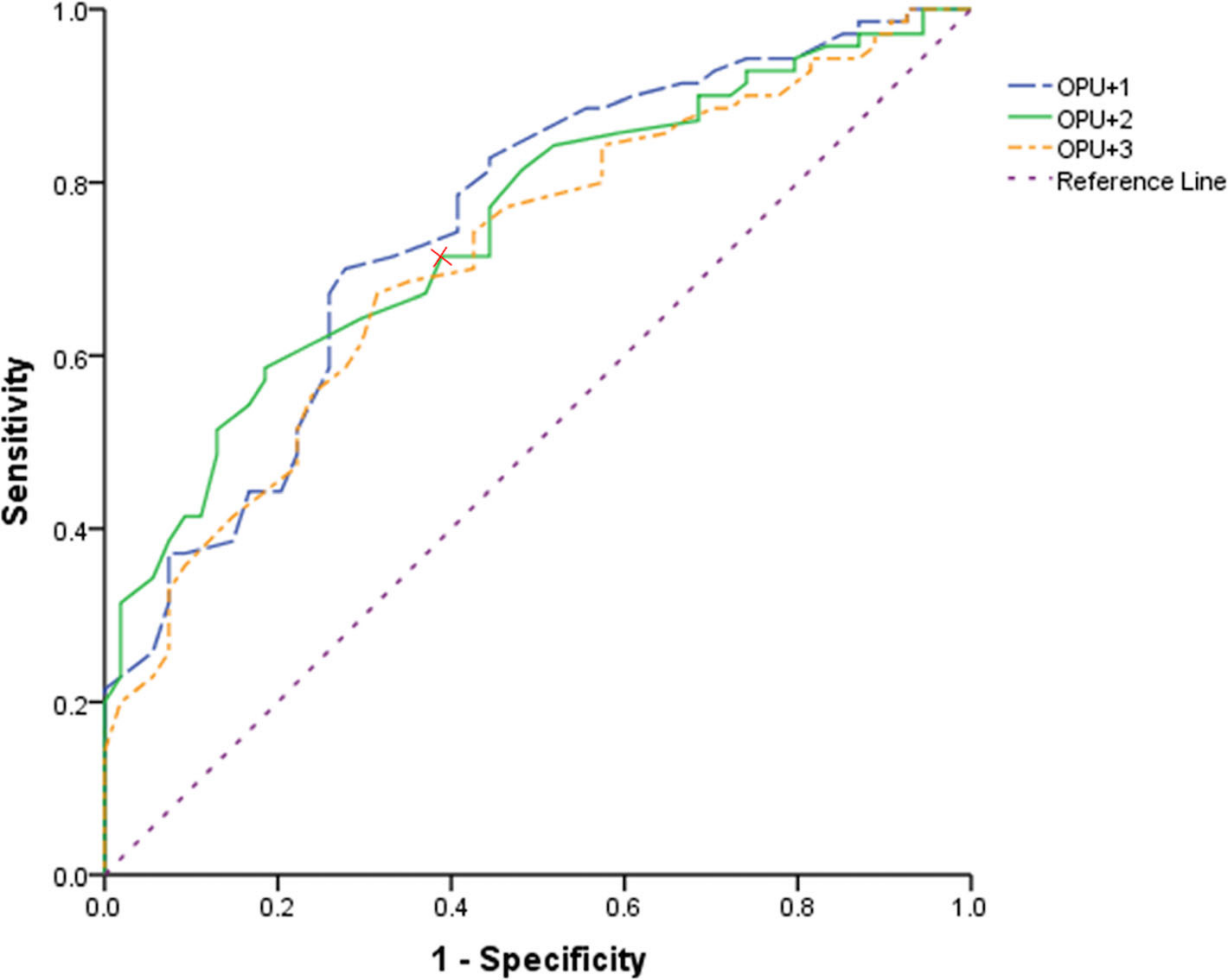
ROC curve of endometrial echogenicity value on OPU + 1,2,3 for successful clinical pregnancy. The areas under the ROC curve were 0.738(95%CI:0.656–0.819), 0.765(95%CI: 0.688–0.842), 0.714(95%CI:0.624–0.804) respectively on OPU + 1, OPU + 2 and OPU + 3. Endometrial echogenicity value on OPU + 2 had the most predictive value, and the cutoff value was 76.5%. The sensitivity was 61.3% and the specificity was 82.0%

**Fig. 5:**
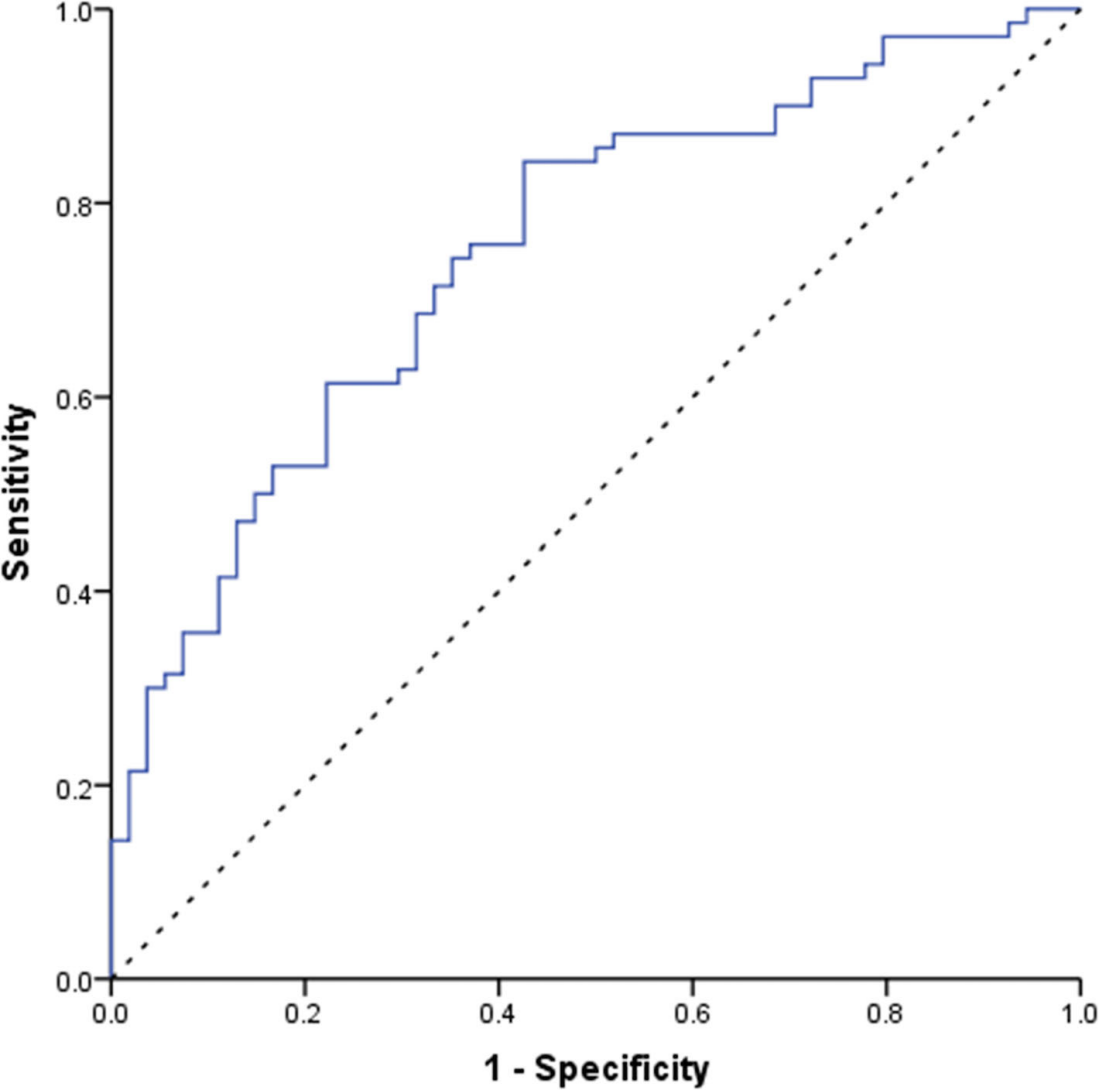
ROC curve of the combination of endometrial echogenicity value on OPU + 2 and thickness on OPU + 3. The areas under the ROC curve was 0.751 (95%CI:0.665–0.836)

The publishers apologise for this error.
